# Brazil’s Regulatory Context for Using New Approach Methodologies (NAMs) on the Registration of Products

**DOI:** 10.3389/ftox.2022.903027

**Published:** 2022-07-22

**Authors:** Izabel Vianna Villela, Miriana da Silva Machado

**Affiliations:** InnVitro Pesquisa and Desenvolvimento—Support and Management in Toxicology, Porto Alegre, Brazil

**Keywords:** new approach methodologies, NAMs, ANVISA, registration, safety assessment

## Abstract

New Approach Methodologies (NAMs) are any non-animal-based approaches that can provide information in the context of chemical hazard and safety assessment. The goal is to develop information with equivalent or better scientific quality and relevance than that provided by traditional animal models. Starting with ethical issues, these approaches are gaining regulatory relevance in different global agencies. Since 2008, with the enactment of the Arouca Law—the first Brazilian legislation dedicated to laboratory animals, NAMs are gathering pace in Brazil’s regulations. Specific regulations from different sectors include the acceptance of these new methods. However, some regulation is controversial about what is needed to address specific toxicological endpoints. The resulting regulatory uncertainty induces companies to keep on adopting the traditional methods, slowing NAM’s development in the country. This work brings a perspective on the regulatory acceptance of NAMs in Brazilian Legislation for the registration of pharmaceuticals, medical devices, food/supplements, and agrochemical products. This text discusses the main issues of NAM adoption for each specific regulation. Therefore, legal acceptance of NAMs results in Brazil is still a process in progress. A collective effort including regulators, industry, contract research organizations (CROs), and the academic environment is needed to build regulatory confidence in the use of NAMs.

## Introduction

Previously referred to as alternative methods to animal experimentation, new methodological approaches (NAMs) refer to any method, protocol, change, and set of strategies that reduce the number of animals, reduce the severity of the procedure, or replace the use of laboratory animals, following the 3 Rs principle (Wambaugh et al., 2019). The move from classical animal tests to NAMs is an international trend that began with the discussion of the ethical issues regarding the use of animals for cosmetics testing. However, as science developed, it became clear that the use of NAMs goes beyond ethical issues, and its central premise is to improve the prediction of toxic effects in humans (Parish et al., 2020). An evaluation strategy based on NAMs integrates *in silico*, *in chemico*, and *in vitro* approaches to understand the initial mechanistical endpoints that lead to the adverse effects observed *in vivo*. These approaches are not designed to become direct substitutes for *in vivo* methods but to bring better evidence about adverse effects to different target species (which is usually humans; however, under the premise of the development of veterinary products and the assessment of ecotoxicity, other species may become the main concern).

Limitations of each approach, for example ADME (administration, distribution, metabolism, and excretion), for toxicodynamic understanding and false positives in *in vitro* results should be carefully observed.

The NAMs concept permeated all safety evaluation fields and is reflected in how regulatory toxicology is approached, consequently changing the applicable regulations.

**FIGURE 1:**
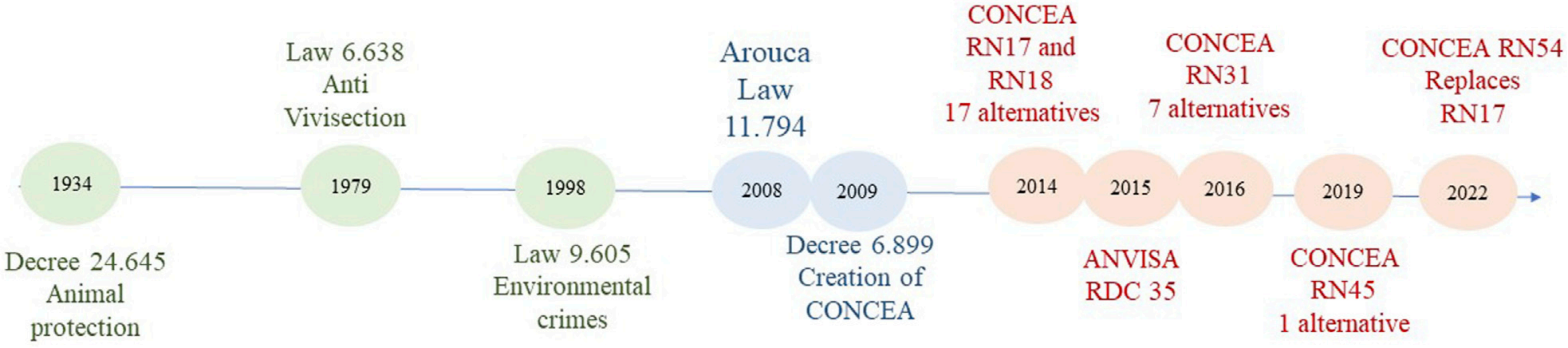
Brazilian Animal Testing and New Approach Methodologies Regulation timeline.

Figure 1 resumes animal experimentation/NAMs legislation development in Brazil. Since 1934, Brazil has established legislation regarding animal protection, including the animal protection decree (BRASIL, 1934), anti-vivisection law (BRASIL, 1979), and environmental crime law (BRASIL, 1998). However, animal experimentation only came into focus with the enactment of the Arouca Law (Law 11.794/2008), which changed how Brazil conducts animal experimentation (BRASIL, 2008). This law focuses on the use of animals in teaching and research activities and regulates their use, management, and assay conditions, focusing heavily on animal welfare. The fundamental point of the Arouca Law was the creation of the National Council for Animal Experimentation Control CONCEA (Conselho Nacional de Controle de Experimentação Animal). This democratic entity includes representatives of Scientific Institutions and Legally Constituted Animal Protection Societies. CONCEA has appointed a permanent committee dedicated to alternative methods, responsible for the most important resolutions in the field. In September 2014, CONCEA issued the Normative Resolution 17/2014. This resolution defines the need to approve alternative tests by CONCEA and, after 5 years of their approval, the approved methodologies become a priority to be applied in toxicological evaluation (CONCEA, 2014a). Normative Resolution 18 (RN 18), also issued in 2014, approved, for the first time, methods considered as alternatives to their original versions. This resolution approved 17 methods, including alternatives for seven toxicological outcomes (toxicological outcome: effect monitored by a toxicological study) (CONCEA, 2014b). After those, Resolution 31 from 2016 to 45 from 2019, they have approved eight other methods (CONCEA, 2016, 2019). In 2022, a Normative Resolution 54/2022 replaced NR 17/2014, with more complete definitions and the possibility of using internationally recognized methods, even if not officially approved by CONCEA. According to NR54/2022, all approved methods must be used immediately after approval, but a 5-year tolerance was maintained (CONCEA, 2022).

The CONCEA normative resolution 17/2014 (CONCEA, 2014a) became even more important following the issue of the Board Resolution (Resolução da Diretoria Colegiada) RDC N. 35/2015 by the Brazilian Health Regulatory Agency ANVISA (Agência Nacional de Vigilância Sanitária), which delineates the acceptance of the alternative methods to animal experimentation recommended by CONCEA (BRASIL, 2015b). This act conferred regulatory significance to the methods approved by CONCEA; it was about their acceptance by the Council and the regulatory agency, thus impacting all the productive sectors regulated by ANVISA.

In order to promote the use of NAMs in Brazil, initiatives such as the Network for Alternative Methods RENAMA (Rede Nacional de Métodos Alternativos) formed in 2012 and relaunched in 2021 (BRASIL, 2021a); the Brazilian Center for the Validation of Alternative Methods (BraCVAM), officially launched in 2013 (Presgrave et al., 2016); and the Mercosur’s Platform for Alternative Methods (Premasul), responsible for almost 30 training courses, were vital.

Even with the constant legislation evolution, specific legislation does not always meet the exact requirements; keeping the regulatory acceptance of NAMs is a constant issue. Challenges include meeting the legislation requirements of different sectors, the quality of presented data (study reports and literature review), availability of certified facilities (GLP or ISO17025), the correct interpretation of data, and the transparency of the decision-making process.

Thus, this work aims to discuss the Brazilian regulatory context and challenges of using NAMs in safety assessment for the registration of pharmaceuticals, medical devices, food/supplements, and agrochemical products.

## Pharmaceuticals

Like other global health agencies, safety evaluation is necessary to register new synthetic, biological, and herbal medicines at ANVISA (BRASIL, 2010, BRASIL, 2011, BRASIL, 2014, BRASIL, 2017, BRASIL, 2018b). In the post-registration phase, toxicological evaluation may also be necessary, such as drug impurity qualification (BRASIL, 2015c).

According to ANVISA legislation related to the registration of pharmaceutical products, the safety evaluation of new drug products includes nonclinical and clinical studies (BRASIL, 2010, BRASIL, 2011, BRASIL, 2013, BRASIL, 2014, BRASIL, 2017, BRASIL, 2018b; BRASIL, 2019b; BRASIL, 2019d). Harmonized to ICH (International Council for Harmonisation of Technical Requirements for Pharmaceuticals for Human Use), EMA (European Medicines Agency), and FDA (United States Food and Drug Administration)-related guidelines, acute toxicity, dose-repeated toxicity, reproductive toxicity, genotoxicity, local tolerance, carcinogenicity, safety pharmacology, immunotoxicity, and toxico/pharmacokinetics are the minimal nonclinical endpoints to be investigated. The studies should be performed according to validated and recognized guidelines, such as OECD (Organization for Economic Co-operation and Development) protocols, and in Good Laboratory Practice (GLP) certified facilities. ANVISA recommends replacing *in vivo* studies with validated and internationally accepted alternative methods (BRASIL, 2013, BRASIL, 2019c).

Regarding drug impurities, depending on the impurity levels detected in the stability analysis of the drug and the product posology, toxicological evaluation may also be needed to demonstrate the product’s safety. This process, named impurity qualification, involves at least mutagenicity and general toxicity (BRASIL, 2015a; 2015c). Aligned to ICH M7 assessment and control of DNA reactive (mutagenic) impurities in pharmaceuticals to limit a potential carcinogenic risk (ICH, 2017), ANVISA recommends *in silico* methods as the first approach for mutagenicity evaluation (BRASIL, 2015a). Depending on the result profile, quality, and confidence level, a confirmatory *in vitro* test (such as the Ames test) should be carried out. Thus, based on literature data, *in silico*, and *in vitro* results, the impurity is classified among 1 and 5 classes. For general toxicity, generally, a 28-day or 90-day repeated dose toxicity test in rodents is needed. As for new drug development, studies must be performed under OECD and GLP technical and quality requirements.

## Medical Devices

According to RDC No. 546:2021 (BRASIL, 2021b), medical devices must be designed and manufactured in such a way that the characteristics and performance are guaranteed, with particular attention to:

1) selection of materials used, particularly in terms of toxicity and, where applicable, flammability; and
2) compatibility between materials and the biological tissues, cells, and body fluids, considering the intended purpose of the medical product.

Thus, products that come into direct contact with the patient need to be evaluated for preclinical and clinical safety. Biological evaluation (nonclinical safety) is performed according to the ISO 10993 guideline series. The previous version of ISO 10993-1 (Evaluation and Testing Within a Risk Management Process) presented a biological evaluation based on a list of required toxicological endpoints—including animal tests for most of them. However, the 2018 version brought a new concept: the only mandatory approach is physicochemical characterization (ISO, 2018). All toxicological endpoints should be addressed according to expert evaluations and considering the existing data. The toxicological rationale should be well described and properly sustained by a literature review and, when needed, by appropriate assay reports. The consistency and clarity of information are crucial; presenting a well-explained and documented rationale to integrate the technical dossier is a crucial step. However, the regulator does not accept poor literature reviews, and the companies understand that the agencies are more likely to authorize tested devices and usually go straight to animal testing. In this case, regulatory uncertainty is not related to conflicting information but to the correct interpretation of the guidelines.

Specific assays must be performed only when a complete literature review is insufficient to secure biocompatibility. The ISO 10993 series is constantly updated, including NAMs in evaluating specific endpoints. Considering CONCEA’s new regulation (RN 54/2022; CONCEA, 2022), the release of a new approach for medical devices by ISO should be enough to be used in place of animal testing. So, it should not bring regulatory uncertainty. On the other hand, new tests should be used in the scope they were validated and carried out by competent laboratories (under GLP or ISO17025) (ISO, 2018), and their results should be adequately evaluated, discussed, and included in the biocompatibility report. Most of the problems stem from not correctly performed experiments or not adequately discussed results. In this case, scientific rigor during all the processes is the main element for regulatory acceptance.

## Food/Supplements

To register new foods or new ingredients for human consumption in Brazil, applicant companies must submit to ANVISA a series of information about the product, mainly according to resolutions No. 16/1999 and No. 17/1999 (BRASIL, 1999a; 1999b) and related resolutions. ANVISA classifies as “new foods or new food ingredients” those substances that do not have a history of use in Brazil or that are already consumed but that will be used in quantities much higher than those currently found in foods used in the regular diet (BRASIL, 1999a).

Among the necessary information, there is evidence of safety, which aims to protect the population’s health and reduce possible risks associated with the consumption of the product. The safety assessment involves complete risk analysis, including information about characterization, history of use data, toxicological data, intended use, and exposure, that must compose the Technical Scientific Report. The report must be aligned to Guide No. 23/2019 (BRASIL, 2019a) for food and food ingredients. Safety evaluation of probiotics is also included in other legislation, such as RDC 241:2018 (BRASIL, 2018a), and has a specific Guide No. 21/2021 (BRASIL, 2021c). Both guides do not detail specific protocols but generally indicate endpoints. Both documents suggest using animals in specific endpoints for toxicological assessment, mainly because it is needed to derive a safety value such as ADI (acceptable daily intake). In addition, the guidelines mentioned mainly *in vivo* methods for the evaluation of most toxicological endpoints. In this context, the uncertainty in using NAMs is related to extrapolating a safety value not based on animal results, a topic that is not consolidated yet.

## Agrochemicals

The evolution of agrochemical toxicity regulation in Brazil was extensively reviewed by da Silva et al. (2020). A significant change in the toxicological evaluation and classification approach came in 2019 when ANVISA launched a new regulation, the RDC 294:2019 (BRASIL, 2019c). The updated approach is based on the Globally Harmonized System of Classification and Labelling of Chemicals (GHS) criteria, bringing ANVISA closer to the international requirements (BRAZIL, 2019c).

The regulations directly mention the preference to use alternative methods approved by CONCEA, OECD, or authorities with similar regulatory exigences. Nevertheless, the strict requirements related to the classification raise doubts about the acceptability of NAM results.

We can take, for example, skin irritation and corrosion. OECD Guidelines TG431 *In Vitro* Skin Corrosion: reconstructed human epidermis (RHE) test method (OECD, 2019) and TG439 *In Vitro* Skin Irritation: reconstructed human epidermis test method (OECD, 2021) are validated and widely used to identify GHS category 1—corrosive, category 2—irritant, and no classified—no irritant. The only gap in classification using these guidelines would rely on category 3—mild irritant. Both assays were approved by CONCEA and are available in Brazil under GLP conditions; according to ANVISA’s regulation RDC 35:2015, they would be the first choice to test this endpoint. However, the categorization criteria described in RDC 294:2019 are based only on the number of animals and dermal damage (BRASIL, 2019b). In this context, companies would prefer to use animal tests to address RDC 294:2019 specific requirements even with well-established alternative guidelines.

## Conclusion/Perspectives

Brazil is at the cutting edge in Latin America regarding the regulatory acceptance and use of NAMs. In the last two decades, ANVISA and CONCEA have released several guidelines about NAMs’ acceptance in the toxicological evaluation of products (which are summarized in Table 1 and Figure 1).

**TABLE 1:** National Health Surveillance Agency (ANVISA) legislations and guides related to the nonclinical evaluation of pharmaceuticals, medical devices, food, and agrochemical products.

Product type	Legislation/guideline	Subject	Reference
Pharmaceuticals	Director’s Collegiate Resolution (RDC) n° 24, from June 14th, 2011.	Sets parameters for the registration of specific medicines.	BRASIL (2011)
	Guide for conducting nonclinical toxicology and pharmacological safety studies necessary for drug development, from 31 January 2013. Version 2	Guide for conducting nonclinical toxicology and pharmacological safety studies necessary for drug development	BRASIL (2013)
	Director’s Collegiate Resolution (RDC) n° 26, from May 13th, 2014.	Sets parameters for the registration of herbal medicines, and the registration and notification of traditional herbal medicines.	BRASIL (2014)
	Guide n° 04, from 4 December 2015. Version 1	Guide for obtaining the degradation profile and identification and qualification of degradation products in medicines	BRASIL (2015a)
	Director’s Collegiate Resolution (RDC) n° 242, from July 26th, 2018.	Regulates the registration of vitamins, minerals, oral amino acids and proteins, classified as specific medicines.	BRASIL (2018b)
	Director’s Collegiate Resolution (RDC) n° 53, from 4 December 2015	Sets parameters for the notification, product identification, and qualification degradation in drugs with substances synthetic and semi-synthetic actives, classified as new, generic, and similar, and takes other measures	BRASIL (2015c)
	Guide n° 22, from June 17th, 2019. Version 1	Guide for conducting non-clinical studies necessary for herbal medicines and traditional herbal medicines development.	BRASIL (2019b)
	Efficacy and Safety Analysis Roadmap for Synthetic Drug Registration Evaluation, from 26 May 2019	Efficacy and Safety Analysis Roadmap for Synthetic Drug Registration Evaluation	BRASIL (2019d)
Medical devices	Director’s Collegiate Resolution (RDC) n° 546, 30 August 2021	Establishment of the essential requirements for the efficacy and safety of medical devices	BRASIL (2021c)
Food	Resolution n° 17, 30 April 1999	Basic Directives to Food Risk and Safety Assessment	BRASIL (1999b)
	Guide n° 23, from 9 August 1999. Version 1	Guide for proving the safety of food ingredients	BRASIL (2019a)
	Guide n° 21, from 6 May 2021. Version 2	Guide for processual petition of evaluation of probiotics for use in food	BRASIL (2021a)
Agrochemicals	Director’s Collegiate Resolution (RDC) n° 294, 31 July 2019	Establishment of criteria for the toxicological evaluation and classification of agrichemicals, its components, and related products	BRASIL (2019c)

NAMs are accepted for registering pharmaceuticals, medical devices, food/supplements, and agrochemical products at ANVISA if carried out following validated protocols, under the scope for which they were validated, and under quality control conditions (GLP or ISO17025). However, it is important to keep in mind that every strategy has its own limitations, so it is crucial to understand what can be concluded from each experiment. A regulatory accepted equivalency of approaches is usually not a direct replacement for one test to another. All information is needed to complement the approach and the understanding of the adverse effect should be provided.

However, some regulations generate conflicting interpretations by the regulator or regulated sectors. In many cases, it is not the NAMs themselves that are not accepted by ANVISA, but a lack of robust results and flaws in the execution and description stages of the assay included in the registration process is what causes such a negative response. Regulators need to feel certain about the acceptance of any results, and this comes from the use of validated methods with adequately designed experiments, considering the specificities of the tested product. At the same time, the regulated sector needs to feel certain about how the regulator will interpret data.

The main points regarding the regulatory acceptance of NAMs can be summarized as follows:

- Use of validated methods within the scope they were validated.
- Rigorous conduct of the experiments, preferred by GLP laboratories.
- Complete test reports, including at least reference material, historical laboratory data, number of replicates, and responsible research.
- Complete literature review, with searching and detailed methods and analyses.
- Robust scientific analysis of results by experts in the subject with scientifically supported conclusions.
- Transparence of the regulatory evaluation process, making clear how each result is being evaluated.

Transparency is a fundamental point in the complete process, from developing and validating methods to decision-making. Divergent perspectives/expectations, the lack of transparency, and the strength of data are the main barriers to building trust in NAMs. These issues are not only from Brazilian reality and are constantly being discussed internationally.

To address these points, sharing knowledge is vital. Training initiatives such as PremaSul (Regional Platform for Alternative Methods for the Use of Experimental Animals) have delivered a great result in disseminating approved methods through the academy and regulated sector. Now, we need more interaction between sectors. The creation of working groups mediated by scientific societies and technical associations, such as RENAMA (National Network of Alternative Methods), bringing academy, industry, and regulators to the same table to discuss the best approach to the different endpoints, will be the next step to harmonize needs and expectations. The collaboration among different stakeholders will provide transparency to the whole process, building trust in NAMs and consequently regulatory certainty.

## Data Availability

The original contributions presented in the study are included in the article/Supplementary Material; further inquiries can be directed to the corresponding author.
